# A novel compound heterozygous mutation in *CYP11B2* (p.A153P and p.Q337*) associated with primary hypoaldosteronism

**DOI:** 10.3389/fmed.2026.1740124

**Published:** 2026-03-11

**Authors:** Jianmei Yang, Lingyu Li, Yan Sun, Jie Jiang, Xiangbo Xie, Weiwei Xu, Chen Chen

**Affiliations:** 1 Department of Pediatric Endocrinology, Shandong Provincial Hospital Affiliated to Shandong First Medical University, Jinan, Shandong, China; 2 Shandong University of Traditional Chinese Medicine, Jinan, Shandong, China; 3 Department of Neonatology, Shandong Provincial Hospital Affiliated to Shandong First Medical University, Jinan, Shandong, China; 4 Endocrinology, SBMS, Faculty of Medicine, The University of Queensland, St Lucia, QLD, Australia

**Keywords:** aldosterone synthase deficiency, CYP11B2 gene, hyperkalemia, hypoaldosteronism, hyponatremia

## Abstract

**Objective:**

Congenital aldosterone synthase deficiency (ASD), a subset of primary hypoaldosteronism caused by *CYP11B2* mutations, is characterized by hyponatremia, hyperkalemia, and elevated plasma renin (with normal cortisol production). This article focused on the clinical and genetic analysis of aldosterone synthase deficiency type II to achieve a deep understanding of the ASD pathophysiology.

**Methods:**

The clinical biochemical data and whole exome genetic data of a newborn ASD patient were analyzed. The pathogenicity of the novel mutations was predicted using the Result Prediction Software (REVEL). Three-dimensional structures of the mutated gene coded protein was calculated. Steroid hormone level was measured by mass spectrometry. Literatures of all related reports were collected using the HGMD database and Pubmed to analyze the similarity and difference of the novel mutations.

**Results:**

The male neonate, born at 26^+2^ weeks of gestation via emergency cesarean section, presented with poor respiratory function and low Apgar scores. Laboratory tests revealed severe hyponatremia, hyperkalemia, elevated plasma renin, and decreased cortisol levels. The mass spectrometry results indicated that the aldosterone level was very low. Two mutations in CYP11B2 gene were delected, including *c.1009C > T* and the novel mutation *c.457G > C*, with pathogenicity by affected protein structure and function. A total of 82 mutation sites including *c.457G > C* on CYP11B2 gene have been reported so far. The patient was treated with 9α-fluorohydrocortisone, and biochemical and plasma renin levels returned to normal after in 1 month. After 3 months, cortisol levels were normalized. The patient is currently under long-term follow-up.

**Conclusion:**

Persistent hyponatremia necessitates thorough evaluation to rule out hypoaldosteronism, where genetic testing facilitates early definitive diagnosis. Long-term clinical follow-up remains critical for individualized treatment optimization. In this study, we reported the first identification of a novel *CYP11B2* mutation (*p.A153P*) and confirmed its genetic association with the classic hypoaldosteronism phenotype, expanding the mutational spectrum of *CYP11B2* and providing novel insights into the genetic basis of ASD. This newly identified mutation expands the spectrum of mutations in primary hypoaldosteronism.

## 1 Introduction

Congenital aldosterone deficiency (ASD) is a rare genetic endocrine disorder caused by *CYP11B2* mutations, which impair aldosterone biosynthesis (1). The incidence rate is less than 1/1,000,000 (2). It was first linked to *CYP11B2* defects by Russell in 1963, presenting with neonatal hyponatremia, hyperkalemia, metabolic acidosis, and failure to thrive (1). ASD is classified into two subtypes based on steroid profiles: Type I features undetectable aldosterone, elevated 18-OHDOC, and low 18-OHB; while Type II presents with low aldosterone, normal/high 18-OHB, and a reduced 18-OHB/aldosterone ratio. The current standard treatment for ASD involves lifelong fludrocortisone replacement, dietary sodium supplementation, and regularly monitoring blood electrolytes and pressure to prevent hyperkalemia and hypertension. Consistent with established practice, this case study applied the standard protocol. Notably, the efficacy of standard protocol in a patient with the rare *p.A153P CYP11B2* mutation was validated, reinforcing the applicability of current guidelines to this specific population. The diagnosis of ASD is often challenging due to its nonspecific symptoms similar to other pathological conditions, and requires a combination of biochemical tests, imaging studies, and genetic analysis (3, 4). To date, eighty-two *CYP11B2* mutation variants have been reported. This study reported a patient with hypoaldosteronism carrying a *CYP11B2* compound heterozygous mutations (including a new mutation *p.A153P*) and explored the association between genotype and phenotype of the case.

## 2 Materials and methods

### 2.1 Patient and ethical approval

#### 2.1.1 Ethical approval

The Institutional Human Ethics Review Board at Shandong Provincial Hospital affiliated to Shandong First Medical University approved this study (SWYX:NO.2023-532). The participant or legal guardians of the participant were given a written information to obtain the signed consent to participate in the study. This study conformed to the provisions of the Declaration of Helsinki.

#### 2.1.2 Patient

In 2023, a newborn boy, delivered by emergency cesarean section in Shandong Provincial Hospital. He was admitted due to “low vitality 21 min after birth.” G1P1, gestational age 26^+2^ weeks, extremely preterm, birth weight 800g. After birth, he exhibited weak respiration, with Apgar scores of 4 at 1 min, 6 at 5 min, and 8 at 10 min. The patient experienced severe respiratory distress and required intubation and mechanical ventilation. The parents of this patient were physically healthy and had a nonconsanguineous. The clinical evaluation, baseline and dynamic hormonal levels, and genetic analyses were performed and obtained with signed consent form by the parents.

### 2.2 Clinical observations

Initial and sequential plasma levels (ionogram and hormonal profile) in patient were measured. Adrenocorticotropic hormone (ACTH), Cortisol (COR), Renin, aldosterone, creatinine, and ionogram (K^+^, Na^+^, Cl^–^) were measured. Laboratory reference ranges were as follows: ACTH: 7.2–63.3 pg/ml, COR: 166–507 nmol/L, Renin: 3.8–38.8 pg/ml, aldosterone: 40–310 pg/ml, creatinine: 19–44 μmol/L, K: 3.5–5.5 mmol/L, Na: 135–145 mmol/L, Cl: 98–110 mmol/L. All hormones were measured by chemiluminescent methods (Roche, Basel, Switzerland) following the manufacturer’s instructions. Renin, aldosterone, creatinine, ionogram (K Na Cl) were measured in the hospital laboratory.

### 2.3 Steroid hormone determination

Steroid hormone determination was measured using mass spectrometry. Plasma samples were subjected to protein precipitation using methanol, and then the supernatant was purified and enriched for the analytes through solid-phase extraction. Impurities were eluted with n-hexane and 10% acetonitrile, and finally, the analytes were eluted with 90% acetonitrile and pure water, resulting in samples containing the analytes and their internal standards. The processed samples were analyzed for signal collection and quantification using high-performance liquid chromatography-tandem mass spectrometry.

### 2.4 Genome sequencing

A volume of 3–5 mL of peripheral venous blood was obtained from the proband as well as his parents. The DNA extracted from the peripheral blood underwent whole exome sequencing (WES). Following the manufacturer’s instructions, the exons derived from the genomic DNA of the patient were fragmented, ligated, amplified, and purified. Subsequently, the SeqCap EZ Med Exome Enrichment Kit (Roche NimbleGen) was utilized to capture exons and their adjacent regions from all known genes. Post-capture amplification and purification were performed, after which the DNA library was constructed using the Illumina HiSeq system.

The sequence data were aligned to the human genome reference version 19 (hg19) using NextGene V2.3.4 to ensure adequate coverage and an appropriate depth of mean reads within the targeted regions. Information regarding conserved nucleotide bases and amino acids, as well as the frequencies within normal populations (sourced from the 1,000 Genomes Project, ExAC, dbSNP DNA, and other locus-specific databases), predictions of biological functions, and data from The Human Gene Mutation Database (HGMD), ClinVar, and Online Mendelian Inheritance in Man (OMIM) were gathered through NextGene V2.3.4. Variants were screened in accordance with established guidelines. The pathogenicity of variants was assessed following the criteria set forth by the American College of Medical Genetics (ACMG) for the interpretation of sequence variants, as published in 2015, using the nomenclature established by the Human Genome Variation Society (HGVS) (5).Sanger sequencing was used to verify the variants in the proband revealed by WES, and to test the co-segregation of variants in the family. Genome sequencing was completed in collaboration with GrandOmics.

### 2.5 Bioinformatic analysis

The spatial structure of the *CYP11B2* protein and the affected protein regions after mutations were demonstrated. Prediction of three-dimensional protein structures based on the three-dimensional structure of mutant *CYP11B2* was achieved using I-TASSER software (6)^1^ The PyMOL Viewer software was used to visualize the effects of altered residues on the protein structure models. Additionally, we used the following result prediction softwares (REVEL), including SIFT,^2^ PolyPhen2 HVAR,^3^ Mutation Taster,^3^ MutationAssess,^4^ and FATHMM^5^ were used.

### 2.6 Literature comparison

To comprehensively review the mutations in patients with ASD type II, searches for primary studies of ASD were conducted on PubMed and WANFANG MED ONLINEMEDLINE on June 25, 2025. Mesh terms “aldosterone synthase deficiency type II or aldosterone synthase deficiency type 2” and “*CYP11B2* mutation” were applied for the literature search. A total of 13 patients were included, and their general information, biochemistry, genetics, and prognosis were compiled into a table and analyzed (Table 1).

**TABLE 1:** The general situation, biochemical characteristics, genetics, and prognosis of reported cases.

Patients	Gender	Age of onset	Symptoms	COR (nmol/L)	Renin (pg/mL)	Aldosterone	K (mmol/l)	Na (mmol/l)	Cl (mmol/l)	Gene mutation	Prognosis	PMID
1	Male	5 months	Poor feeding, failure to thrive, repeated vomiting, hyponatremia	274.3 (59.9–480)	-	0.166nmol/l (0.39-2.44)	-	-	-	c.977C > A (p.T326K) and c.523_525delAAG (p.K175del)	A good long-term prognosis	24,694,176
2	Female	41 weeks	Poor feeding, failure to thrive	-	-	-	-	–	-	T185I–T498A	-	12,788,848
3	Female	23 days	Poor feeding, failure to thrive,	18.46 μg/dL normal	31 ng/mL/h (0.32-1.84)	235.4 pg/mL (30022100321900)	-	-	-	p.T185I (ACC-ATC) (c.554C > T) (g.7757C > T)	Good clinical effect on body growth and psychomotor development.	32,857,717
4	Male	39 weeks	Vomiting, failure to thrive	62ng/ml normal	3760 ng/L (1.7-23)	< 0.03 ng/mL (0.03–0.82)	-	-	-	Homozygous missense mutation c.554C > T(p.T185I)	-	22,565,077
5	Male	4 weeks	Failure to thrive, vomiting, hypotonia, hyperkalemia, and hyponatremia	-	> 75 ng/mL/h (4-12)	3.7 ng/dL (20-120)	-	-	-	-	-	6,991,942
6	Male	8 weeks	Failure to thrive, spitting up	9151 ng/dL (80-1500)	15.2 ng/mL/h (1-4)	19ng/dl (4-31)	5.2	121	93	-	Poor	7,485,152
7	Male	1 year 10 months	Failure to thrive, dehydrated	-		-	-	114	-	-	Good clinical effect	2,044,581
8	Male	3 months	Failure to thrive, infection, hypovolaemic shock	Normal	-	-	5.4	122	-	-	Good clinical effect	2,044,581
9	Female	2 weeks	Vomiting, diarrhea, and severe dehydration	23 μg/dL (3-21)	-	1.8 ng/dL (5-80)	5.0	136	–	–	–	3,510,001
10	Male	6 weeks	Failure to thrive, dehydrated	5.4 μg/dL (3-21)	-	6.9 ng/dL (5-80)	7.3	125	–	–	–	3,510,001
11	Male	1 month	Failure to thrive, repeated vomiting	-	45.5 ng/mL/h (1-4)	-	6.0	122	–	–	–	1,709,913
12	Male	3 months	Infection, hypovolaemic shock	-	-	130 pmol/L (280–2,200)	–	–	–	–	–	9,625,333
13	Male	10 days	Poor sucking, repeated vomiting	5.7 μg/dL (3–21)	50 ng/mL/h (1–4)	6 ng/dL (20–120)	5.9	127	–	–	–	1,601,005

COR, cortisol. The normal range of K is 3.5–5.5 mmol/L, Na 135–145 mmol/L, Cl 98–110 mmol/l.

## 3 Results

### 3.1 Clinical features

A male neonate, born at 26^+2^ weeks (birth weight 800 g) of gestation via emergency cesarean section, presented with poor respiratory effort and low Apgar scores (scored 4, 6, or 8 at 1, 5, and 10 min after delivery in infant). Initial assessments revealed severe respiratory distress requiring intubation and mechanical ventilation. The infant appeared as a preterm neonate with poor responsiveness, and perioral cyanosis. Under intubation, breath sounds were consistent, with no dry or moist rales noted. Heart beats were strong and regular, with no detected murmurs detected in the valve auscultation areas. The abdomen was soft, with the umbilical cord intact and free of bleeding or discharge. Reduced muscle tone was noted in the limbs, with diminished primitive reflexes.

### 3.2 Laboratory tests and treatment

The first symptom of the child after birth was hyponatremia, with blood sodium levels of 114 mmol/L, blood potassium levels of 7.43 mmol/L, and blood chlorine levels of 84.4 mmol/L. The child was treated with oral concentrated sodium and intravenous sodium supplementation. After treatment, the effect was poor. At the same time, it was found that cortisol decreased from 124 to 29 nmol/L and ACTH decreased from 18.4 to 7.73 pg/ml. Oral sodium salt was discontinued, and 30 ml of intravenous physiological saline and 1.2 mg of hydrocortisone were administered intravenously once a day. After treatment, the blood sodium level increased to 132 mmol/L, blood potassium level was 5.67 mmol/L, blood chlorine level was 102 mmol/L, cortisol level was 703 nmol/L, and ACTH level was 7.95 pg/ml. After the condition improved, the patient switched to oral physiological saline and acetic acid chloride cortisone tablets. Examination showed that cortisol level decreased to 46.7 nmol/L after 193 nmol/L, and ACTH level increased to 13.4 pg/ml, but the blood sodium level remained stable. After the diagnosis of *CYP11B2* mutation through genetic examination, oral saline was stopped and 9a fluoro-hydrocortisone was added. Afterwards, cortisol levels steadily increased to 159 nmol/L, and blood sodium remained stable at 134–139 mmol/L. The original treatment plan was maintained and continued.

### 3.3 Bioinformatic analysis

**FIGURE 1:**
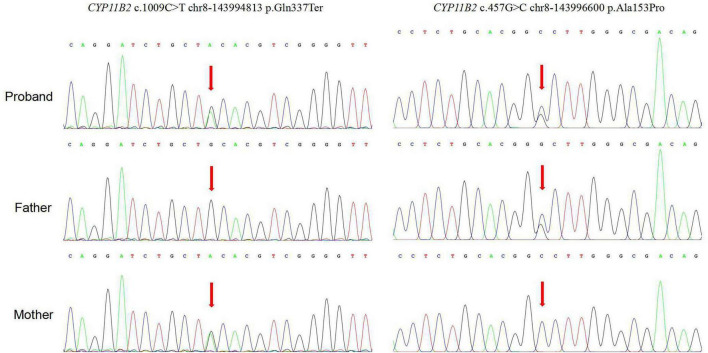
Genetic testing results of the patient’s family.

**FIGURE 2:**
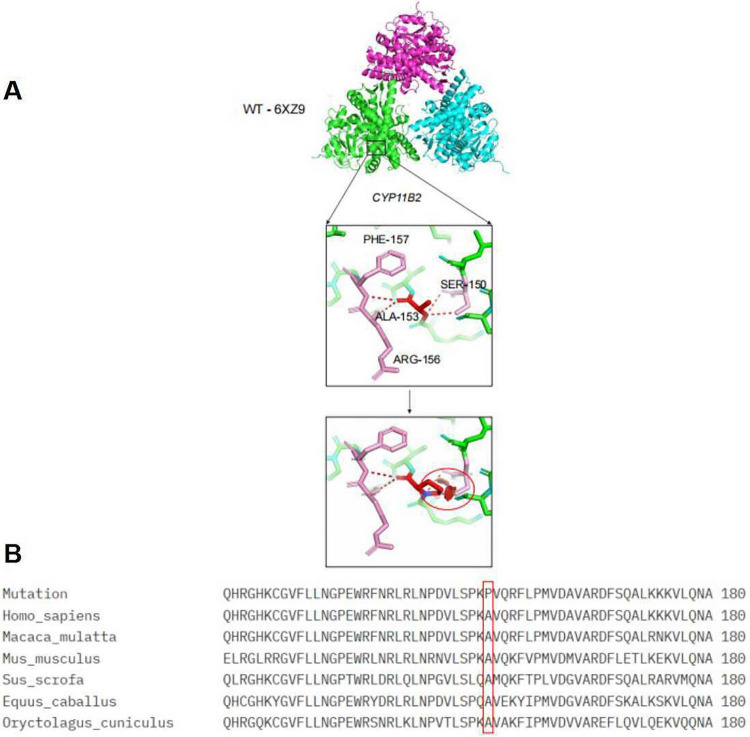
**(A)** WT-6XZ9 represents a three-dimensional structural model of the wildtype *CYP11B2* protein, designated 6XZ9 in the Protein Data Bank (PDB). *CYP11B2* represents the gene. PHE-157, SER-150, ALA-153, ARG-156,the numbers represent amino acid residues of PHE (phenylalanine), SER (serine), ALA (alanine), ARG (arginine). **(B)** Mutation: indicates that this section displays sequence information related to the ALA-153 mutation. Homo_sapiens: indicates that this sequence originates from humans. Macaca_mulatta: indicates that this sequence originates from macaques (a non-human primate species). Mus_musculus: indicates that this sequence originates from mice (a commonly used model organism). Sus_scrofa: indicates that this sequence originates from wild boars/domestic pigs. Equus_caballus: indicates the sequence originates from horses. Oryctolagus_cuniculus: indicates the sequence originates from European rabbits/domestic rabbits. QHRGHKCGVFLNLNG…. (long string): this is an amino acid sequence (represented by single-letter abbreviations), displaying the primary structure of the *CYP11B2* protein fragment corresponding to this species. 180: indicates the amino acid residue number corresponding to the right-aligned end of the amino acid sequence (i.e., residue 180), used for localization.

The patient had compound heterozygous pathogenic variants in the CYP11B2 gene. The mother was heterozygous for the c.1009C > T chr8-143994813 p.Q337*, which had been previously reported (7) and the father was heterozygous for the *c.457G > C chr8-143996600 p.A153P*, which was a new pathogenic variant (Figure 1). The pathogenicity of novel mutation *c.457G > C* was predicted using multiple software tools, with a combined score of pathogenicity (Table 2). A conservation analysis discovered that the 153 rd alanine (A) of the CYP11B2 gene (*NP_000489.3*) was conserved across different species. The *p.A153P* (exon3) mutation site affects its polar interaction with serine at position 150, which may affect protein function (Figure 2).

**TABLE 2:** Pathogenicity of two mutations in pediatric patients.

Variant locus	Protein change	HGMDpro database report status	Family origin	GnomAD Frequency (East Asian)	ACMG Evidence	Interpretation conclusion
c.1009C > T	p.Q337*	Reported as pathogenic variant (DM), associated with Aldosterone synthase deficiency, PubMed ID:26936515	Mother	0.000163	PVS1 + PM2_Supporting + PM3	Pathogenic
c.457G > C	p.A153P	No report available	father	0	PM2_Supporting + PM3	Variant of Uncertain Significance (VUS)

*Terminator codon.

The c.*1009C > T* variant carried by the proband has been clearly identified as a pathogenic variant, with strong evidence chains of PVS1 (nonsense variant triggering nonsense mediated mRNA degradation), PM3 (trans distribution with another pathogenic variant), and PM2 (extremely low population frequency). The other variant *c.457G > C*, although separately classified as a clinically unknown variant (VUS), is trans distributed with pathogenic variants and has supporting evidence for PM2 (population frequency of 0) and PM3 (presence of pathogenic variants in the trans position), which conforms to the rule of “one definite pathogenic and one supporting pathogenic in a compound heterozygous pair.” Final classification conclusion: According to the ACMG guidelines, this complex heterozygous variant combination should be classified as a pathogenic complex heterozygous variant, which can clearly explain the pathogenesis of aldosterone synthase deficiency in the proband (Table 2).

### 3.4 Steroid profiling analyzes

Plasma steroid profiling was conducted after a 1-week cessation of therapy when the subject was 1 year old (Table 3). The mass spectrometry results indicated that 11-deoxycorticosterone, corticosterone, and 18-hydroxycorticosterone were all within normal or slightly above normal concentration levels for children of the same age group, but the aldosterone level is very low. During the administration of fludrocortisone at a daily dosage of 66 μg, the subject exhibited normal developmental milestones, electrolyte levels, and blood pressure.

**TABLE 3:** Plasma steroid profile of the patient.

Steroid profile	Numerical value	Unit	Reference range
Pregnenolone	372.5	pg/mL	Not applicable
Progesterone	0.03	ng/mL	≤ 0.5
11-deoxycorticosterone	298.7	pg/mL	< 300.0
Corticosterone	5.06	ng/mL	0.18–19.70
18-Hydroxycorticosterone	657.49	pg/mL	100–6,700
Plasma Aldosterone	62.2	pg/ml	≤ 400.0
17a-Hydroxypregnenolone	0.20	ng/mL	0.35–7.12
17-Hydroxyprogesterone	0.07	ng/mL	>6.30
11-Deoxycortisol	169.5	pg/mL	<3,440
21-Deoxycortisol	0.7	pg/mL	<50
Cortisol	35.6	ng/mL	50–250
Cortisone	12.9	ng/mL	5.0–39.0
18-hydroxycortisol	241.1	pg/mL	318–1,666
18-oxocortisol	0.3	pg/mL	Not applicable
Dehydroepiandrosterone	0.03	ng/mL	< 2.3
Dehydroepiandrosteron e sulfate	4.1	ng/mL	110-1200
Androstenedione	9.3	pg/mL	< 690.0
Testosterone	8.5	pg/ml	50.0–4,000.0
Dihydrotestosterone	3.6	pg/mL	≤ 1,200.0
11-hydroxyandrostenedione	85.3	pg/mL	370–3,508
11-ketoandrostenedione	2.7	pg/mL	Not applicable
11-hydroxytesterone	4.0	pg/mL	Not applicable
11-ketoTestoterone	43.9	pg/mL	Not applicable
Epi-Testoterone	0.82	pg/mL	Not applicable
Androsterone	9.61	pg/mL	Not applicable
Epi-Androsterone	1.47	pg/mL	Not applicable
T/DHT	2.36	Not applicable	Not applicable
11OHAD/AD	9.17	Not applicable	Not applicable
11KAD/AD	0.29	Not applicable	Not applicable
11OHT/T	0.47	Not applicable	Not applicable
11KT/T	5.16	Not applicable	Not applicable
Estrone	0.0	pg/mL	≤ 16.0
β-Estradiol	3.1	pg/mL	≤ 13.0
Estriol	0.00	pg/mL	≤ 180.0

### 3.5 Management and follow-up

This patient was followed up closely since his diagnosis in endocrinology clinics and has demonstrated a favorable clinical and laboratory response to mineralocorticoid therapy. Regular monitoring of ACTH, cortisol, renin, aldosterone, creatinine, and biochemical parameters was implemented to adjust medication dosages accordingly. On May 21, 2024, after cortisol levels returned to normal (319 nmol/L), hydrocortisone acetate was discontinued and only treatment with fluoxetine was maintained. The most recent follow-up on January 14, 2025, showed normal parameters with the infant measuring 78 cm in height and weighing 9 kg. The patient will be followed up in the future to maintain a healthy physiological condition (Table 4). In summary, the patient was treated with 9α-fluorohydrocortisone and sodium supplementation for 1 month, to achieve biochemical maker and plasma renin levels returned to normal. After 3 months, cortisol levels were normalized, and the patient is currently under long-term follow-up schedule.

**TABLE 4:** Initial and sequential plasma levels (ionogram and hormonal profile) in China patient with congenital hypoaldosteronism due to aldosterone synthase deficiency

Date	Height (cm)	Weight (kg)	Age (days)	ACTH (pg/ml)	COR (nmol/l)	Renin (pg/ml)	Aldosterone (pg/mL)	Creatinine (μmol/L)	K (mmol/L)	Na (mmol/L)	Cl (mmol/L)	Therapy
29/04/2023	-	-	0			-	-	-	7.43	125.2	112.2	-
31/05/2023	-	-	32	18.4	124	-	-	-	4.6	114	83.2	Oral 20% NaCl, ivdrip0. 9%NaCl
02/07/2023	-	-	64	7.73	29	-	-	-	-	-	-	Hydrocortisone1 + 0.5 mg
08/09/2023	-	3.4	132	13.4	68.4	>500	224.52	250	5.5	134	103	Hydrocortisone 1.5+1 +1 mg, 5 mL 0. 9%NaCl q3h
20/10/2023	-	3.4	174	11.3	159	56.24	68.92	8.7	4.81	138	106	Hydrocortisone 1.5 mg, Fludrocortisone 0.033 +0.033 mg
04/01/2024	63	6.8	250	30.2	251	92.73	81.76	18.7	4.83	139.4	106.8	Hydrocortisone 1.5 mg, Fludrocortisone 0.033 +0.033 mg
01/03/2024	67	-	306	12	271	65.72	74.87	18.2	4.76	137	104	Hydrocortisone 1 mg, Fludrocortisone 0.033 + 0.033 mg, Oral 0.9% NaCl 10 mL
21/05/2024	70	-	387	12.2	319	96.65	107.32	18.9	5.08	137.1	104.7	Fludrocortisone 0.033 + 0.033 mg Oral 0.9% NaCl 10 mL
30/07/2024	71	7.5	457	78.3	155	126.7	95.37	26	5.1	137.5	106.2	Fludrocortisone 0.033 + 0.033 mg
13/09/2024	71	7.5	502	29.3	216	64.63	81.24	31.8	4.78	138.3	106.6	Fludrocortisone 0.033 + 0.033 mg
14/01/2025	78	9	625	31.8	208	3.46	68.54	21.2	4.76	141.5	108	Fludrocortisone 0.033 + 0.033 mg
24/06/2025	82.5	9.3	786	30.9	540	140.54	176.39	28.6	4.05	137.5	102.3	Fludrocortisone 0.033 + 0.033 mg

ACTH, adrenocorticotropin; COR, cortisol.

### 3.6 Literature review

To review the clinical information and genetic data in patients with ASD type II, reports of primary studies were thoroughly searched in PubMed and WANFANG MED Onlinemedline databases. A total of 13 papers were recorded (8–17), and available clinical information and genetic data were tabulated and analyzed (Table 1).

**FIGURE 3:**
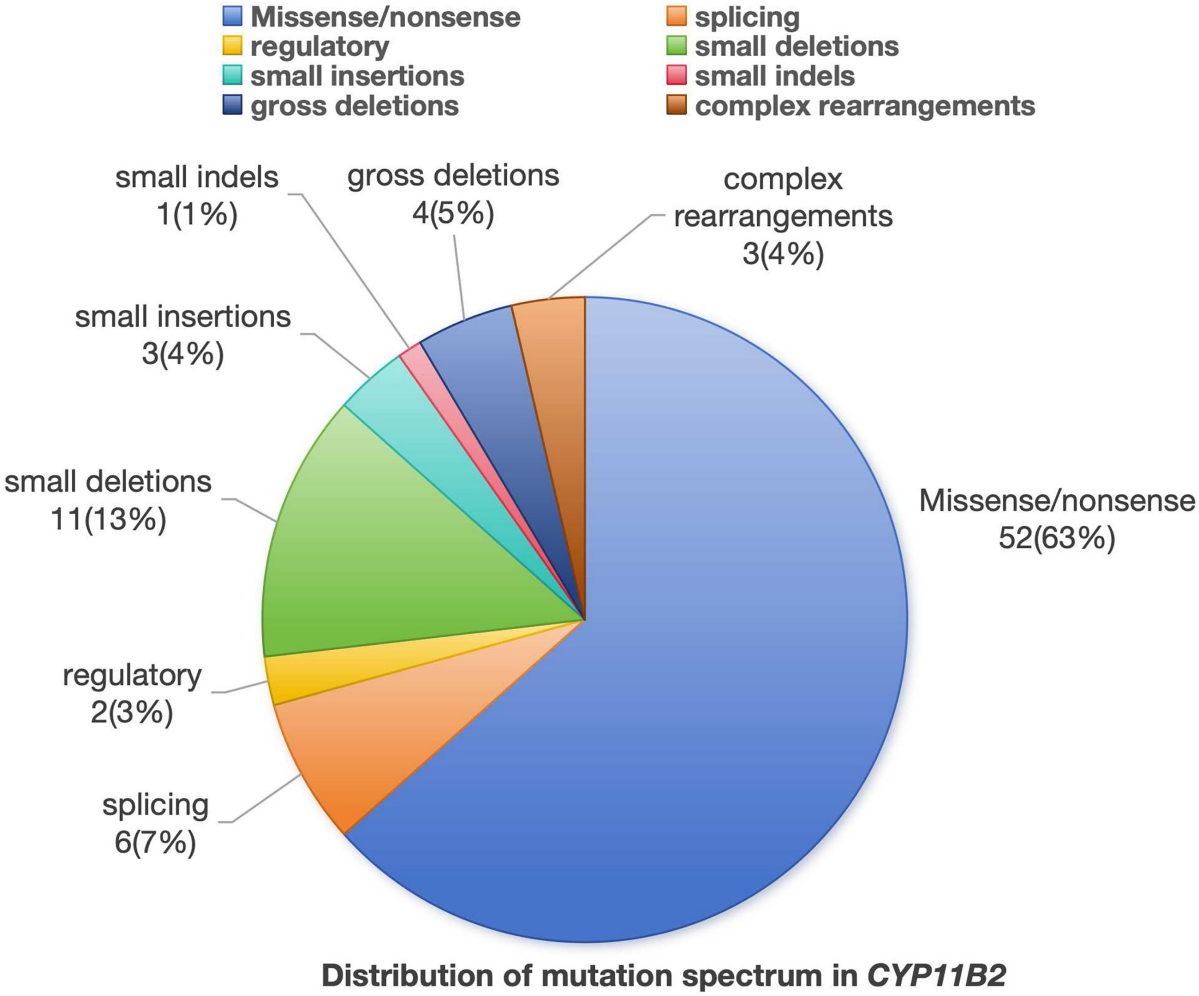
All mutation sites related to the CYP11B2 gene.

A total of 82 mutation sites related to the CYP11B2 gene have been reported, including 52 missense/nonsense mutations (63%), 11 small deletions (13%), 6 splicing mutations (7%), 4 gross deletions (5%), 3 complex rearrangements (4%), 3 small insertions (4%), 2 regulatory (3%), and 1 small indels (1%) (Figure 3 and Supplementary Table 1).

## 4 Discussion

In this study, a novel variant in the CYP11B2 gene was identified in a boy of ASD type II. This patient was the second ever reported CYP11B2 gene mutation-ASD type II case in China and the fourteen reported CYP11B2 gene mutation-ASD type II case internationally (5–14). This discovered variant has never been reported in literature. The significant deteriorating property of this novel mutation was verified by bioinformatic analysis. The prognosis of the patient after 9α-fluorohydrocortisone and sodium supplementation treatment was determined in a follow-up regular check showing an excellent recovery in clinic observation and blood hormone and biochemical assay. Such outcome was particularly rare, and this case highlighted the importance of considering Congenital Hypoaldosteronism in the differential diagnosis of infants presenting with such electrolyte imbalances and failure to thrive. Early recognition and appropriate long-term follow-up management were crucial to prevent life-threatening complications associated with ASD, emphasizing the need for heightened awareness among clinicians when evaluating similar cases (8, 15). Current literature indicates that congenital hypoaldosteronism, particularly with *CYP11B2* mutations, presents a unique clinical symptoms characterized by electrolyte imbalances, growth failure, and potentially life-threatening adrenal crises (3). The genetic underpinnings of this condition reveal a variety of mutations within the CYP11B2 gene, which is crucial for aldosterone synthesis (18).

ASD is caused by mutations in the CYP11B2 gene located on chromosome 8q.24.3, encompassing nine exons and coding for 503 amino acids. The CYP11B2 gene has evolved from the *CYP11B1* gene, with both genes exhibiting high homology within a 40 kb range in the DNA sequence (19). To date, approximately 82 mutations in the CYP11B2 gene have been reported, including missense mutations, nonsense mutations, deletions, insertions, splice mutations, large deletions, and complex rearrangements, with missense and nonsense mutations being the most common (19). *CYP11B2* mutations exhibit ethnic and geographic heterogeneity, with *p*.V386A and *p*.R181W being prevalent in Iranian Jews, while *p*.T185I being a hotspot variant among Greeks and Albanians. Reports of ASD cases in Asia are relatively scarce, and no hotspot variants have been identified. Additionally, the CYP11B2 gene shows significant genetic polymorphism (20). This current case presented a novel mutation, highlighting the complexities associated with ASD, specifically congenital aldosterone deficiency due to mutations in the CYP11B2 gene. This condition was of significant interest due to its rarity and the critical implications it holds for neonatal health. The patient’s diagnosis and subsequent management in this report underline the importance of early identification and tailored therapeutic strategies in improving outcomes for affected individuals. Two mutations in CYP11B2 gene in this patient were validated, including: c.1009C > T chr8-143994813 p.Q337*, and *c.457G > C chr8-143996600 p.A153P* (novel mutation). The *A153P* variant had not been reported in the Clinvar/ HGMD databases for diseases, and there were also no reports of this variant in the normal population databases, indicating that this variant was rare. Although there is no evidence to support its pathogenicity rating, and it is currently classified as a variant of uncertain clinical significance, the clinical presentation is very consistent in this report. ASD type II (aldosterone synthase deficiency type II) is an autosomal recessive genetic disorder caused by mutations in the CYP11B2 gene leading to aldosterone synthesis disorders. Its onset is directly related to genetic defects and has no causal relationship with preterm birth (21). The disturbed renin angiotensin aldosterone system (RAAS) in premature infants may prevent the mature process and aldosterone levels may be reduced and fluctuated, ASD type II patients have the electrolyte imbalances (such as low sodium and high potassium) as the “symptom overlap.” Aldosterone replacement therapy with dexamethasone as the core, supplemented by oral sodium chloride to correct electrolyte imbalance, emergency management of hyperkalemia complications, and adjustment of treatment plan through long-term biochemical monitoring and imaging evaluation to maintain internal environment stability. This was the same as the treatment for children with this disease. Although the gene test for this patient revealed a paternal-derived A153P variant currently defined as of uncertain significance, this mutation may not be excluded from a pathogenic mutation in conjunction with the patient’s medical history of this case. The identification of novel mutations further emphasized the heterogeneous nature of ASD and the necessity for genetic testing to confirm diagnosis and to guide treatment.

In the process of aldosterone synthesis, 11β-hydroxylase (*CYP11B1*) catalyzes the conversion of 11-deoxycorticosterone to corticosterone. It provides the necessary precursor for aldosterone synthesis and is an important upstream enzyme in the aldosterone synthesis pathway. Its deficiency may lead to reduced aldosterone synthesis, resulting in related endocrine disorder diseases. Aldosterone synthase (*CYP11B2*) is the key enzyme in aldosterone synthesis, primarily catalyzing the conversion of corticosterone to aldosterone, and is expressed only in the adrenal zona glomerulosa. The patient’s levels of 11-deoxycorticosterone, corticosterone, and 18-hydroxycorticosterone were all within normal or slightly above normal levels in this patient, but the aldosterone level was very low at 62.2 pg/ml (normal range around 1240 pg/ml). This reflects the improvement in some indicators after treatment with fludrocortisone. The aldosterone synthesis process is influenced by two enzymes, 11β-hydroxylase and aldosterone synthase. The normal concentrations of corticosterone and 18-hydroxycorticosterone in this case indicate that the precursors for aldosterone synthesis were sufficient, but the significantly low aldosterone concentration suggested a deficiency related to a mutation in the CYP11B2 gene that causes a lack of aldosterone synthase.

In managing the patient, the treatment regimen was adjusted based on the genetic findings, transitioning from hydrocortisone therapy to a combination of hydrocortisone and fludrocortisone. This adjustment was particularly notable as it reflected the importance of personalized medicine in the treatment of endocrine disorders, where genetic insights can significantly influenced therapeutic strategies (22). The timely initiation of appropriate steroid replacement therapy was proven effective in alleviating the symptoms associated with adrenal insufficiency and improving the overall quality of patient life. As the patient continues to be monitored, the long-term implications of congenital aldosterone deficiency are becoming clearer. Regular follow-up assessments of hormone levels, growth parameters, and overall health were essential in managing this case and preventing complications. This case illustrated the critical role of continuous monitoring and the potential for positive outcomes with appropriate medical intervention (23).

Future research should focus on expanding our understanding of the genetic variations associated with congenital hypoaldosteronism and their clinical implications. Additionally, studies exploring the psychosocial aspects of living with such a condition, as well as the long-term management strategies, will be invaluable in enhancing clinical practice and patient education. By integrating genetic insights into routine clinical assessments, diagnosis and treatment of neonates suffering from ASD may be improved (24).

## 5 Conclusion

Aldosterone synthase deficiency (ASD) type II shows an important disease related with hereditary hyperkalemia and hyponatremia. Genetic diagnosis achieves an early confirmation, and continuous treatment follow-up is crucial for optimizing treatment and ensuring long-term health. This current study identifies a novel variant in the CYP11B2 gene, enriching the genetic spectrum of *CYP11B2*.

## Data Availability

The raw data supporting the conclusions of this article will be made available by the author, without undue reservation.

## Footnotes

1
https://seq2fun.dcmb.med.umich.edu/I-TASSER

2
http://sift-dna.org

3
http://genetics.bwh.harvard.edu/pph2/

4
https://www.mutationtaster.org/

5
https://fathmm.biocompute.org.uk/index.html
